# Fasting and postprandial regulation of the intracellular localization of adiponectin and of adipokines secretion by dietary fat in rats

**DOI:** 10.1038/nutd.2015.34

**Published:** 2015-11-30

**Authors:** V Olivares-García, I Torre-Villalvazo, L Velázquez-Villegas, G Alemán, N Lara, P López-Romero, N Torres, A R Tovar, A Díaz-Villaseñor

**Affiliations:** 1 Instituto Nacional de Ciencias Médicas y Nutrición Salvador Zubirán, Department Fisiología de la Nutrición, México, D.F., México

## Abstract

**Background/Objective:**

Dietary fat sources modulate fasting serum concentration of adipokines, particularly adiponectin. However, previous studies utilized obese animals in which adipose tissue function is severely altered. Thus, the present study aimed to assess the postprandial regulation of adipokine secretion in nonobese rats that consumed high-fat diet (HFD) composed of different types of fat for a short time.

**Methods:**

The rats were fed a control diet or a HFD containing coconut, safflower or soybean oil (rich in saturated fatty acid, monounsaturated fatty acid or polyunsaturated fatty acid, respectively) for 21 days. The serum concentrations of adiponectin, leptin, retinol, retinol-binding protein-4 (RBP-4), visfatin and resistin were determined at fasting and after refeeding. Adiponectin multimerization and intracellular localization, as well as the expression of endoplasmic reticulum (ER) chaperones and transcriptional regulators, were evaluated in epididymal white adipose tissue.

**Results:**

In HFD-fed rats, serum adiponectin was significantly decreased 30 min after refeeding. With coconut oil, all three multimeric forms were reduced; with safflower oil, only the high-molecular-weight (HMW) and medium-molecular-weight (MMW) forms were decreased; and with soybean oil, only the HMW form was diminished. These reductions were due not to modifications in mRNA abundance or adiponectin multimerization but rather to an increment in intracellular localization at the ER and plasma membrane. Thus, when rats consumed a HFD, the type of dietary fat differentially affected the abundance of endoplasmic reticulum resident protein 44 kDa (ERp44), sirtuin 1 (SIRT1) and peroxisome proliferator-activated receptor-γ (PPARγ) mRNAs, all of which are involved in the post-translational processing of adiponectin required for its secretion.

Leptin, RBP-4, resistin and visfatin serum concentrations did not change during fasting, whereas modest alterations were observed after refeeding.

**Conclusions:**

The short-term consumption of a HFD affected adiponectin localization in adipose tissue, thereby decreasing its secretion to a different magnitude depending on the dietary fat source. Evaluating the fasting serum concentration of adipokines was not sufficient to identify alterations in their secretion, whereas postprandial values provided additional information as dynamic indicators.

## Introduction

Adipose tissue acts as an endocrine organ by secreting hormones and cytokines called adipokines that exert their physiological effects in autocrine and paracrine manners. Among the several adipokines that are secreted by adipose tissue, adiponectin is the most abundant adipokine.^1, 2^

Approximately threefold more adiponectin is secreted compared with most other hormones. Adiponectin has strong insulin-sensitizing, anti-diabetic and anti-inflammatory activities.^2^ In particular, adiponectin reduces the plasma levels of free fatty acids and fat accumulation in the liver, muscle and visceral adipose tissue and prevents pancreatic β-cell apoptosis. Furthermore, it increases hepatic insulin action, mitochondrial function and the rate of glucose-stimulated insulin secretion, all of which improve glucose tolerance.^3, 4^

Adiponectin is secreted into the blood in three major forms: low-molecular-weight (LMW) trimers, medium-molecular-weight (MMW) hexamers and high-molecular-weight (HMW) complexes. The HMW complex is the most active form, and it plays a role in improving insulin sensitivity and protecting against the development of type 2 diabetes (T2D).^2^ The serum levels of adiponectin are negatively correlated with obesity and T2D, and impaired adiponectin multimerization, particularly the selective reduction of HMW complexes, is associated with obesity, insulin resistance, T2D and atherosclerosis.^2^

Dietary management is an alternative way to increase serum adiponectin levels, particularly through the daily intake of fish or ω-3 or fiber supplements.^5, 6, 7, 8, 9^ However, the effect of consuming a high-fat diet (HFD) on fasting serum adiponectin levels remains controversial, and some evidence suggests that the fat source may modulate the serum adiponectin content.^6, 8, 9, 10, 11, 12, 13, 14, 15, 16, 17, 18, 19^

The serum levels of other relevant adipokines, including leptin, retinol-binding protein-4 (RBP-4), resistin and visfatin, are known to increase with obesity.^20^ These adipokines are involved in different aspects of metabolism, such as regulating energy expenditure and food intake, stimulating insulin resistance through the impairment of insulin signaling in muscle, inducing the expression of hepatic gluconeogenic enzymes, regulating insulin secretion and evoking vascular and endothelial dysfunction.^1, 20^

The results of several studies on adipocytes, rodent adipose tissue and human serum samples have suggested that the type of fatty acid or dietary fat that is consumed affects the concentration of several adipokines, including leptin, resistin and visfatin, in addition to adiponectin.^6, 7, 8, 9, 10, 11, 12, 13, 14, 15, 16, 17, 18, 19, 21, 22, 23, 24^ However, the biological mechanisms by which fatty acids regulate adipokine secretion have not been well established. In addition, most of the studies analyzed the serum concentration of secreted adipokines under fasting conditions, and few studies have focused on postprandial changes in serum adipokine levels that are relevant for understanding how dietary components, particularly fatty acids, affect the endocrine functions of adipose tissue. For instance, the regulation of adiponectin secretion and multimerization in response to dietary fat intake has not been explored. Thus, this study aimed to assess the regulation of adiponectin secretion and multimerization during the fasting and postprandial stages in rats fed control diet (CD) or HFD containing different types of dietary fat (coconut, safflower or soybean oil) with distinct proportions of saturated, monounsaturated and polyunsaturated fatty acids for 21 days, before the onset of obesity to avoid the influence of biochemical abnormalities that occur as a consequence of hypertrophic adipose tissue. In addition, we examined whether the fasting and postprandial serum concentrations of other adipokines, such as leptin, RBP-4, resistin and visfatin, were modified by the experimental diets.

## Materials and methods

### Animals and diets

Male Sprague Dawley rats (Harlan Laboratories, México City, México), each weighing ∼190 g, were housed in individual cages at 22 °C with a 12 h light/dark cycle and free access to water. Six groups of 25 rats each were given free access to one of the experimental diets without blinding (Supplementary Table 1) for 3 days and then were fed the corresponding experimental diet on a time-restricted schedule (8 h per day) for 18 more days to synchronize food intake and to prevent potential variability in adipokine secretion associated with changes in the time of food consumption. During this period, the rats developed the habit of consuming their food within the first 10 min after it was provided.

The CD contained 7% w/w fat (15.91% kcal from fat), as recommended by the AIN-93G,^25^ and the HFD contained 21% w/w fat (36.21% kcal from fat). The fat sources for both diets were coconut oil (70% saturated lauric and myristic acids), safflower oil (70% monounsaturated oleic acid) or soybean oil (63% polyunsaturated linoleic and α-linolenic acids)^26^ (Supplementary Table 1). Food intake was determined daily, and body weight was determined every other day. After 21 days of consuming the experimental diets, each of the six groups was subdivided into five groups of five rats each, according to previous studies.^26, 27^ Rats in one of the five subgroups of each experimental diet group were anesthetized using CO_2_ and killed by decapitation after overnight fasting (0 min), whereas the rats in the other subgroups were killed at 30, 60, 90 and 120 min after refeeding. Blood was collected in tubes containing a separating gel and a clot activator (BD, Franklin Lakes, NJ, USA), and serum was obtained by centrifugation at 1000 *g* for 10 min and stored at −80 °C. Epididymal adipose tissue samples were collected and stored at −80 °C until use for RNA or protein extraction, and some samples were fixed in 10% phosphate-buffered formalin for immunofluorescence analysis. The animal protocol was approved by the Animal Care and Research Advisory Committee of the Instituto Nacional de Ciencias Médicas y Nutrición Salvador Zubirán, Mexico City, Mexico.

### Determination of serum biochemical parameters

Serum glucose levels were determined using the glucose oxidase method with a YSI 2700 Select Biochemistry Analyzer (YSI Incorporated, Yellow Spring, OH, USA). Serum cholesterol and triglyceride levels were determined using enzyme-based colorimetric assay kits (DiaSys Diagnostic System, Holzheim, Germany). Serum insulin levels were determined using an ELISA kit (ALPCO, Salem, NH, USA). The above parameters were analyzed in duplicate in each sample. Insulin resistance was evaluated indirectly through the HOMA-IR (homeostatic model assessment of insulin resistance) that was calculated as follows: [fasting glucose (mmol l^−1^) × fasting insulin (μU ml^−1^)]/22.5.^28^

### Determination of serum adipokine levels

The total circulating levels of adiponectin were determined using an ELISA kit (ALPCO), those of leptin were determined using a radioimmunoassay kit (Millipore, St Charles, MO, USA) and those of RBP-4, resistin and visfatin were determined using enzyme immunoassay kits (RayBiotech, Norcross, GA, USA). The levels of each adipokine were determined in duplicate according to the manufacturer's instructions. Total serum adipokine secretion was calculated from the area under the curve (AUC) of the data obtained at fasting and during the refeeding period.

### Quantitative real-time PCR

Total RNA extraction from epididymal white adipose tissue and complementary DNA synthesis were conducted as previously described.^27^ Adiponectin mRNA levels were determined by real-time quantitative PCR using TaqMan Universal Master Mix (Applied Biosystems, Roche, Branchburg, NJ, USA) and TaqMan fluorogenic probes (Rn00595250_m1) (Applied Biosystems, Foster City, CA, USA) and were normalized to the transcript levels of the hypoxanthine phosphoribosyltransferase (HPRT) housekeeping gene (Rn01527840_m1). The relative amount of mRNA was calculated by the comparative CT method^29, 30^ using the fasting CD-soybean oil group as the calibrator sample.

Sirtuin 1 (SIRT1) and peroxisome proliferator-activated receptor-γ (PPARγ) mRNA levels were determined by real-time quantitative PCR using LightCycler 480 SYBR Green I Master Mix (Roche Diagnostics, Indianapolis, IN, USA). The following primer sequences were used to amplify mRNA: SIRT1, 5′-GCAGGTATGGACAGCAAGCA-3′ and 5′-ATATGGACCTATCCGTGGCCTT-3; PPARγ 5′-CGAGTCTGTGGGGATAAAGC-3′ and 5′-CCAACAGCTTCTCCTTCTCG-3′ HPRT, 5′-CTGGTGAAAAGGACCTCTCG-3′ and 5′-GGCCACATCAACAGGACTCT-3′ and cyclophilin, 5′-CGTGGGCTCCGTTGTCTT-3′ and 5′-TGACTTTAGGTCCCTTCTTCTTATCG-3′.

The relative quantification of SIRT1 and PPARγ mRNA was based on the primer efficiency (E=10[−1/slope]) and the crossing point values for each group compared with those of the fasting CD-soybean oil group. Data are presented as the relative transcript levels compared with the HPRT and cyclophilin housekeeping genes, according to the equation for relative expression.^31, 32^ All the PCR assays were conducted in triplicate.

### Western blotting

Proteins were extracted from epididymal white adipose tissue and quantified as described previously.^27^ For standard western blotting, 30 μg of total protein was denatured by heating for 5 min in Laemmli sample buffer containing β-mercaptoethanol (Bio-Rad, Hercules, CA, USA), separated by SDS–polyacrylamide gel electrophoresis using 8% polyacrylamide gels and transferred to polyvinylidene difluoride membranes. The distribution of adiponectin multimers was conducted under nonreducing and non-denaturing conditions using 20 μg of total protein prepared from adipose tissue or 15 μl of serum diluted 1:50 in RIPA buffer. The samples were separated by SDS–polyacrylamide gel electrophoresis using 4–20% polyacrylamide gels (Bio-Rad) and were transferred to polyvinylidene difluoride membranes.

The blotted membranes were blocked for 1 h at room temperature in 5% non-fat dry milk (Bio-Rad) and then were incubated overnight at 4 °C with the following primary antibodies: adiponectin (Millipore, Chemicon Int.; AB3267P; diluted 1:4500), endoplasmic reticulum resident protein 44 kDa (ERp44; Cell Signaling Technology, Danvers, NA, USA; 2886; diluted 1:1000), Ero1-Lα (Santa Cruz Biotechnology, Dallas, TX, USA; sc-98354; diluted 1:1000) or actin (Santa Cruz Biotechnology; sc-1615; diluted 1:1000). After incubation with the secondary antibodies, the blots were developed using the enhanced chemiluminescence method with Immobilon Western Chemiluminescent HRP substrate (Millipore, Billerica, MA, USA), and the labeled bands were visualized using the ChemiDoc MP Imaging System (Bio-Rad). Optical densitometric analysis was conducted using ImageJ 1.42p digital imaging processing software.^33^ The immunoblotting assays were performed 4 times using independent blots.

### Immunofluorescence

Sections of epididymal white adipose tissue were deparaffinized at 60 °C for 20 min, immersed in xylene and rehydrated in a graded series of ethanol solutions and distilled water. The sections were blocked with Background Sniper (BS966, Biocare Medical, Concord, CA, USA) for 30 min at room temperature and then incubated with the following antibodies at room temperature for 2 h: adiponectin (sc-26497; Santa Cruz Biotechnology; diluted 1:100), Na^+^/K^+^ ATPase (sc-48345; Santa Cruz Biotechnology; diluted 1:100) or oxidoreductase-protein disulfide isomerase (sc-376370; Santa Cruz Biotechnology; diluted 1:50). The sections were incubated at room temperature for 1 h with the following secondary antibodies, respectively (1:500 dilution): FITC-conjugated anti-goat (205-095-108, Jackson ImmunoResearch, West Grave, PA, USA), DyLight 549-conjugated anti-mouse (FDM549C, Biocare Medical) or DyLight 405-conjugated anti-mouse (115-475-003, Jackson ImmunoResearch). The images were captured using a Leica DM750 microscope (Leica, Wetzlar, Germany).

### Statistical analysis

The data are presented as mean±s.e.m. The one-sided unpaired *t*-test (using Welch's correction when the variance between the groups was significantly different) was used to analyze the significance of the differences between the rats fed the CD and those fed the HFD containing each dietary oil at fasting and 30 min after refeeding and of the AUC analyses.

One-way analysis of variance followed by Tukey's multiple comparison test was used to analyze the differences in adiponectin secretion among the time points (0, 30, 60, 90 and 120 min) for each dietary condition.

The normality of the data distribution was evaluated using the Kolmogorov–Smirnov test. In all cases, *P*<0.05 was considered significant (GraphPad Prism 5.00, San Diego, CA, USA).

## Results

### Weight gain, energy intake and biochemical variables

After 21 days of consuming the experimental diets, the body weight of the rats in the three HFD groups was higher than that of the rats in the CD groups. However, the rats fed the HFD-coconut oil gained 25% more body weight than those fed the corresponding CD, whereas those fed the HFD-safflower or HFD-soybean oil gained between 6 and 8% more weight than those fed the corresponding CD (Table 1). The food intake by the CD and HFD groups was similar, although the rats fed the HFD ingested almost twofold more energy from fat than those fed the CD, independent of the type of fat consumed (Table 1). The fasting serum glucose and cholesterol levels were not significantly different among the groups, whereas serum insulin levels and the HOMA-IR index were lower in rats fed HFD-coconut oil compared with those fed the corresponding CD. However, no significant differences were observed between the rats fed the HFD or the CD with safflower or soybean oil. Interestingly, the rats fed the HFD had significantly lower fasting serum triglyceride levels compared with those fed the CD, particularly regarding the diets containing safflower or soybean oil (Table 1).

### Adiponectin secretion

**Figure 1:**
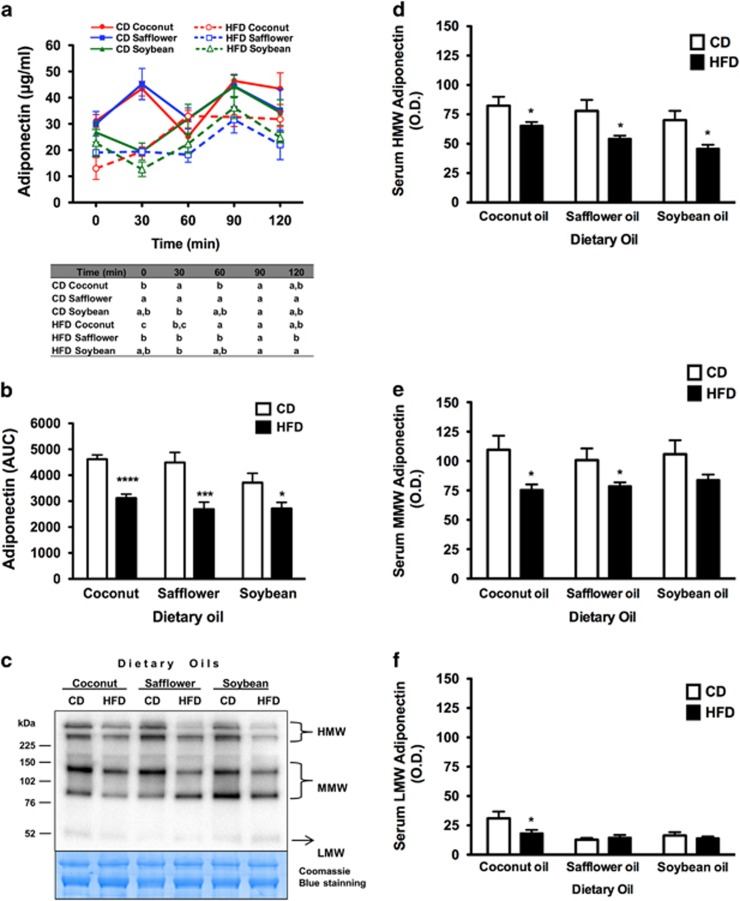
Serum adiponectin. (**a**) Total serum adiponectin concentration at fasting and at 30, 60, 90 and 120 min after refeeding in rats fed the CD or the corresponding HFD containing coconut, safflower or soybean oil for 21 days. The table denotes significant differences among time points (0, 30, 60, 90 and 120 min) for each dietary condition. (**b**) AUC analysis of total serum adiponectin levels at fasting and at 30, 60, 90 and 120 min after refeeding. (**c**) Representative non-denaturing immunoblot of serum adiponectin multimers at 30 min after refeeding in rats fed the CD or the HFD rich in coconut, safflower or soybean oil for 21 days; Coomassie Blue staining served as the loading control. Densitometric analysis of (**d**) HMW, (**e**) MMW and (**f**) LMW adiponectin multimers in serum at 30 min after refeeding in rats fed the CD or the HFD containing coconut, safflower or soybean oil for 21 days. The data are presented as mean±s.e.m. Significant differences at *P<0.05, ***P<0.001 and ****P<0.0001 between rats fed the CD and those fed the corresponding HFD containing each dietary oil. Different superscript letters (a>b>c) indicate significant differences among time points (0, 30, 60, 90 and 120 min) for each dietary condition.

In the rats fed the CD-coconut oil, a biphasic pattern of total adiponectin secretion was observed during the 2 h postprandial stage, and a similar trend was observed in the rats fed the CD-safflower oil. The first peak occurred at 30 min, and the second peak occurred at 90 min after refeeding. In contrast, in the rats that consumed a HFD or CD rich in soybean oil, the peak occurred at 90 min after refeeding (Figure 1a). The total adiponectin concentration at fasting and in the postprandium tended to be lower in the rats fed the HFD compared with those fed the CD (Figures 1a and b). Our data showed that the rats fed the HFD with the three types of fat had lower AUC values than those fed the CD (Figure 1b).

To evaluate the differences in the amount of the three secreted adiponectin multimers based on the type of dietary fat, the serum levels of these multimers were determined at 30 min after refeeding that corresponded to the time of the first peak of serum adiponectin (Figure 1c). Interestingly, the serum level of HMW adiponectin complexes was significantly lower in the rats fed the HFD containing any of the three fats compared with those fed the corresponding CD (Figures 1c and d). However, the serum level of MMW adiponectin was lower only in the rats fed the HFD-coconut or HFD-safflower oil compared with those fed the corresponding CD (Figures 1c and e). The serum level of LMW adiponectin was lower only in the rats fed the HFD-coconut oil compared with those fed the respective CD (Figures 1c and f). The differences in the abundance of the three major multimeric forms of adiponectin in the various dietary groups resembled the differences in total adiponectin levels based on the AUC analysis (Figure 1b).

### Expression and multimerization of adiponectin

**Figure 2:**
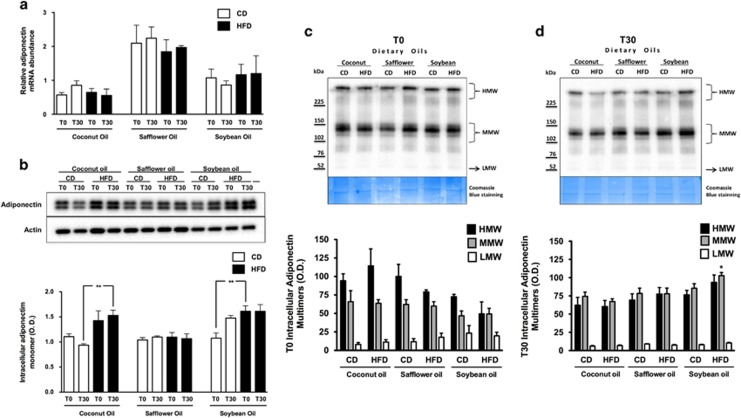
Adiponectin expression in white adipose tissue. (**a**) Relative abundance of adiponectin mRNA normalized to HPRT transcript levels in epididymal white adipose tissue from rats fed the CD or the HFD containing coconut, safflower or soybean oil for 21 days at fasting (T0) and at 30 min after refeeding (T30). (**b**) Representative immunoblot and densitometric analysis of the total intracellular adiponectin monomer content, normalized to actin levels, in white adipose tissue from rats fed the CD or the corresponding HFD containing coconut, safflower, or soybean oil for 21 days at fasting (T0) and at 30 min after refeeding (T30). Representative non-denaturing immunoblot and densitometric analyses of HMW, MMW and LMW adiponectin multimers in epididymal white adipose tissue from rats fed the CD and the corresponding HFD rich in coconut, safflower, or soybean oil for 21 days at fasting (T0) (**c**) or at 30 min after refeeding (T30) (**d**). Coomassie Blue staining was used as the loading control. The data are presented as mean±s.e.m. Significant differences at *P<0.05 and **P<0.01 between rats fed the CD and those fed the corresponding HFD containing each dietary oil.

The observed HFD-induced changes in adiponectin secretion could be partly due to a decrease in adiponectin mRNA levels or to alterations in the adiponectin multimerization process. We did not observe significant differences in adiponectin mRNA levels between animals fed the CD or the HFD containing any of the three fats during fasting or at 30 min after refeeding (Figure 2a). Interestingly, total intracellular adiponectin protein levels tended to be higher in the rats fed the HFD-coconut or HFD-soybean oil compared with those fed the corresponding CD (Figure 2b). Intriguingly, the total intracellular adiponectin protein content did not differ between the rats fed the HFD-safflower oil and those fed the corresponding CD. Interestingly, adiponectin protein content showed an inverse relationship with mRNA abundance for each type and amount of fat, suggesting negative feedback regulation of adiponectin expression (Figures 2a and b).

The abundance of intracellular adiponectin multimers in adipose tissue was determined to assess whether the observed reduction in secreted adiponectin in response to the HFD was the result of disrupted multimerization. Except for a modest increase in MMW adiponectin in the adipose tissue of rats fed the HFD-soybean oil, no significant variations were observed during fasting (Figure 2c) or at 30 min after refeeding (Figure 2d) in the levels of HMW, MMW or LMW adiponectin multimers in adipose tissue between rats fed the CD and those fed the respective HFD, regardless of the type of fat.

### Intracellular localization of adiponectin

**Figure 3:**
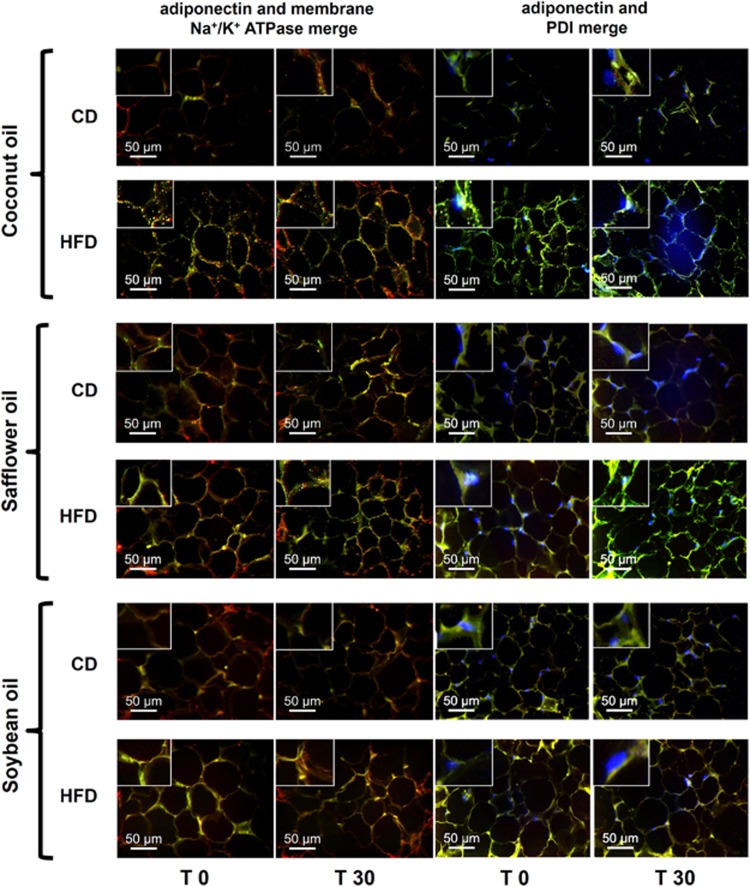
Intracellular adiponectin localization. Representative immunofluorescence images showing the localization (merged image) of adiponectin (green) relative to Na^+^/K^+^ ATPase, which is found at the plasma membrane (red), or to PDI, which is located in the ER (blue), in epididymal white adipose tissue from rats fed the CD or the corresponding HFD containing coconut, safflower or soybean oil for 21 days at fasting (T0) and at 30 min after refeeding (T30); the images were captured using a × 40 objective. At each upper left corner, a magnified image of the representative findings is shown. Scale bar, 50 μm.

The consumption of a HFD could potentially affect adiponectin secretion by altering its intracellular localization. Our data showed increased localization of adiponectin at the plasma membrane of adipose tissue cells from rats fed the HFD compared with those fed the CD, independent of the type of dietary fat, both at fasting and 30 min after refeeding. In particular, in the rats fed the HFD-coconut oil or, to a lesser extent, HFD-safflower oil, adiponectin also formed cytoplasmic aggregates (Figure 3 and Supplementary Figure 1A). Moreover, adiponectin was increased in the endoplasmic reticulum (ER) of adipocytes from animals fed the HFD, particularly those fed coconut and safflower oil (Figure 3 and Supplementary Figure 1B), consistent with the observed decrease in the levels of secreted adiponectin.

### Regulation of adiponectin retention in ER

**Figure 4:**
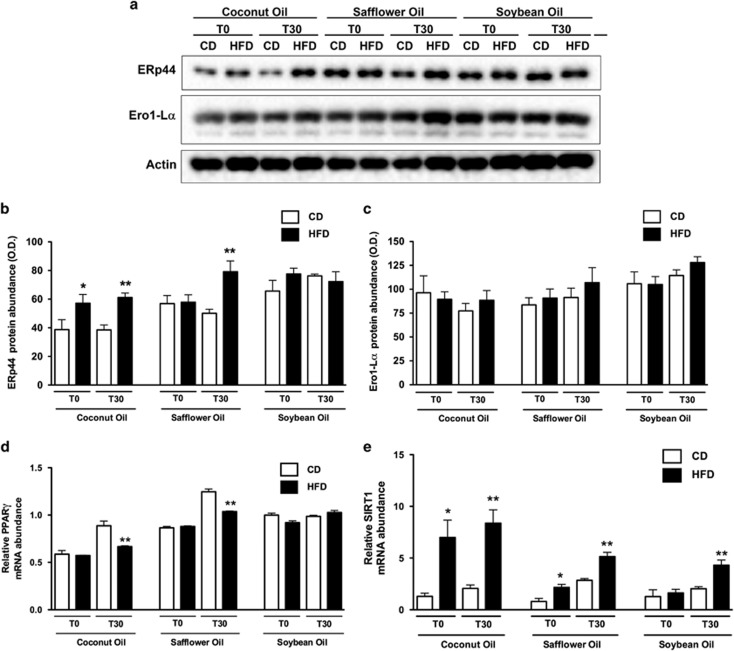
Regulation of adiponectin retention in the ER. Representative immunoblot (**a**) and densitometric analysis of ERp44 (**b**) and Ero1-Lα (**c**) in epididymal white adipose tissue from rats fed the CD or the corresponding HFD containing coconut, safflower or soybean oil for 21 days at fasting (T0) and at 30 min after refeeding (T30). Relative mRNA abundance of PPAR**γ** (**d**) and SIRT1 (**e**) normalized to HPRT and cyclophilin transcript levels in epididymal white adipose tissue from rats fed the CD or the HFD containing coconut, safflower or soybean oil for 21 days at fasting (T0) and at 30 min after refeeding (T30). The data are presented as mean±s.e.m. Significant differences at *P<0.05 and **P<0.01 between rats fed the CD and those fed the corresponding HFD containing each dietary oil.

Adiponectin is retained in the ER lumen in adipocytes by binding to the thiol ERp44 and is released for secretion by the ER membrane-associated oxidoreductase Ero1-Lα.^34, 35, 36^ PPARγ represses ERp44 expression but induces Ero1-Lα expression.^35, 37^ In addition, the NAD-dependent deacetylase SIRT1 has been shown to repress PPARγ by docking to its negative cofactors.^38, 39^ Thus, SIRT activation and/or PPARγ inhibition in adipose tissue decreased adiponectin secretion through the induction of ERp44 and/or the repression of Ero1-Lα (Supplementary Figure 2). Interestingly, our data showed that the abundance of ERp44 increased significantly in the adipose tissue of the rats fed the HFD-coconut oil at fasting and after refeeding or the HFD-safflower oil only after refeeding, but no differences were observed with soybean oil (Figures 4a and b). Accordingly, PPARγ mRNA abundance was significantly decreased after refeeding in the rats fed the HFD-coconut or HFD-safflower oil (Figure 4d). In addition, SIRT1 mRNA levels were significantly increased in rats fed the HFD compared with those fed the CD. The strongest induction was observed in rats fed the HFD-coconut oil in both the fasting and refed states, followed sequentially by rats fed the HFD-safflower oil and finally those fed the HFD-soybean oil only after refeeding (Figure 4e). These results are in agreement with the increased localization of adiponectin in the ER (Figure 3) and the reduction in adiponectin secretion (Figure 1). In contrast, Ero1-Lα protein levels were not modified by diet (Figures 4a and c).

### Fasting and postprandial serum concentration of other adipokines

**Figure 5:**
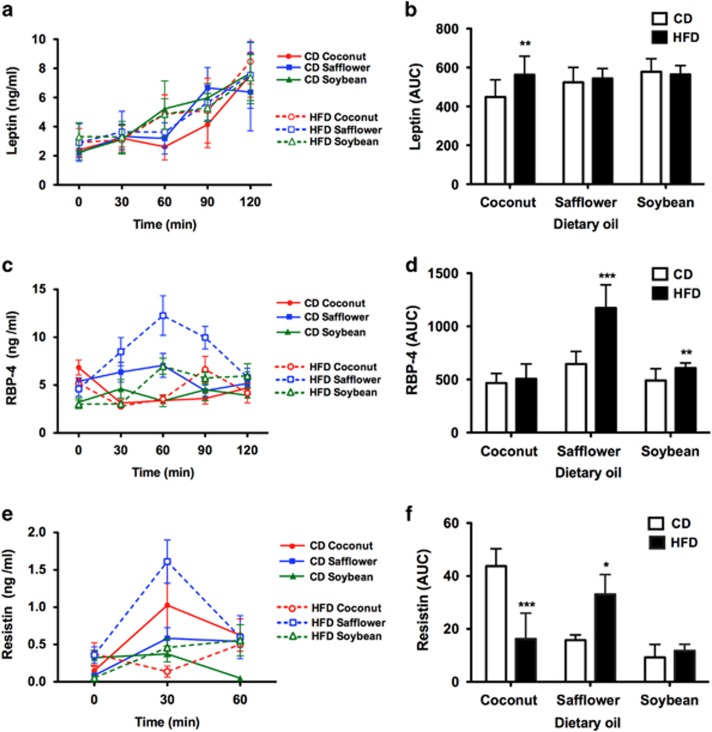
Serum levels of leptin, RBP-4 and resistin. Serum levels and AUC values for leptin (**a**, **b**), RBP-4 (**c**, **d**) and resistin (**e**, **f**) at fasting and at 30, 60, 90 and 120 min after refeeding in rats fed the CD or the HFD rich in coconut, safflower or soybean oil for 21 days. The data are presented as mean±s.e.m. Significant differences at *P<0.05, **P<0.01 and ***P<0.001 between rats fed the CD and those fed the corresponding HFD containing each dietary oil.

The serum leptin concentrations in the six experimental diet groups were similar at fasting (2.66±0.11 ng ml^−1^) and at 2 h after refeeding (7.50±0.26 ng ml^−1^; Figure 5a). Nonetheless, the leptin AUC analysis revealed a significant increase in only the rats fed HFD-coconut oil compared with those fed the corresponding CD (Figure 5b).

The pattern of RBP-4 secretion changed depending on the type and concentration of fat consumed by the rats. However, a similar concentration was reached in all of the groups at the end of the 2 h postprandial period. The rats fed the CD or HFD containing coconut or soybean oil showed modest changes over time. However, the rats fed HFD-safflower oil showed the greatest increase in RBP-4 concentration at 60 min after refeeding; these levels decreased rapidly during the following 60 min (Figure 5c), resulting in an AUC that was significantly higher compared with the other groups (Figure 5d).

The serum resistin concentration was detectable only within the first hour after refeeding. The serum resistin concentrations at fasting and at 60 min after refeeding were similar among the experimental groups. However, at 30 min after refeeding, the serum resistin concentration depended on the diet. The rats fed the HFD-coconut oil had the lowest serum resistin levels, whereas the rats fed the HFD-safflower oil showed the highest concentration (Figure 5e), resulting in parallel changes in the AUC values (Figure 5f). Serum visfatin levels were undetectable in all of the rats except those fed the HFD-safflower oil at 30 min after refeeding (0.65±0.37 ng ml^−1^).

## Discussion

Alterations in circulating adipokines levels are associated with the development of metabolic syndrome and T2D. Here, we reported that the type and amount of dietary fat modulated the circulating levels of adiponectin and other relevant adipokines in rats, particularly during the postprandial period, before the onset of significant weight gain and the development of obesity-related insulin resistance.

We found that adiponectin secretion was particularly sensitive to the composition and amount of dietary fat. Thus, one of the primary events that occurred before the onset of obesity or insulin resistance was impaired adiponectin secretion in response to the dietary fat composition.

In our study, adiponectin secretion was lower in rats that consumed the HFD than in those that consumed the CD, primarily at 30 min after refeeding. In addition, the secretion patterns of particular multimers were altered, but the decrease in adiponectin multimers was due not to an alteration in mRNA abundance or to the multimerization process, but rather to an accumulation of adiponectin at the plasma membrane and ER. Interestingly, the magnitude of this effect was dependent on the type of dietary fat (Supplementary Figure 2).

SIRT1 and PPARγ regulate adiponectin expression in adipocytes as well as its secretion by modulating the expression of several ER chaperones involved in adiponectin post-translational processing.^40, 41^ Specifically, it has been reported that inhibiting SIRT1 and/or activating PPARγ increases HMW adiponectin secretion.^35^ In accordance with this finding, HFD-fed rats showed an increase in SIRT1 expression and a decrease in PPARγ expression, leading to increased ERp44 content that was associated with increased localization of adiponectin at the ER and reduced adiponectin secretion, particularly regarding the HMW complex (Supplementary Figure 2). These results support the hypothesis that in adipose tissue, SIRT1 acts in concert with lipid-regulating transcription factors to adapt adipocyte metabolism to changes in nutrient availability.^39^

Interestingly, studies have shown that a HFD reduces SIRT1 expression.^42^ In mice made obese by the consumption of a HFD (17 weeks) and in *db/db* diabetic mice, the serum adiponectin concentration and adiponectin mRNA levels in adipose tissue are markedly lower than those in lean mice.^40^ In the present study, we did not observe changes in adiponectin mRNA abundance, but SIRT1 mRNA content was higher in the HFD-fed animals. The discrepant results appear to depend on the duration of HFD consumption, but our study aimed to assess differences in adipocyte metabolic functions in response to a HFD before the onset of significant weight gain. Although Ero1-Lα is involved in the release of adiponectin from the chaperone ERp44 and is regulated at the expression level by SIRT1 and PPARγ,^35, 37^ we did not observe a change in Ero1-Lα expression.

The fasting serum adiponectin concentration decreases in mice fed a HFD containing different oils, including lard, olive, sunflower or canola oil, for 10 weeks, and this change is accompanied by insulin resistance.^11^ These authors reported that only mice that consumed lard as the fat source developed more subcutaneous and visceral (epididymal, retroperitoneal and perirenal) adipose tissue mass compared with those fed a CD, particularly by an increase in hypertrophic adipocytes in epididymal fat.^11^ In fact, gonadal visceral adipose tissue is one of the largest adipose depots in rodents and is specifically increased in animals fed a cafeteria diet.^43^ Interestingly, fish oil increases adiponectin mRNA abundance in a dose-dependent manner in epididymal but not in subcutaneous adipose tissue in mice.^44^ However, future research is necessary to understand how dietary fat affects the regulation of adiponectin secretion in adipose depots other than the epididymal depot.

Because low circulating adiponectin concentrations are associated with insulin resistance and metabolic syndrome,^2, 3^ the identification of dietary constituents that alter serum adiponectin levels, not only at fasting but also during the postprandium, is of great clinical interest. We demonstrated that postprandial adipokines stimulation is influenced by specific dietary components, in particular by the fatty acid composition of specific oils. This finding is supported by other studies that showed that treating 3T3-L1 adipocytes and human primary adipocytes with long chain ω-3 fatty acids increases adiponectin release^45, 46^ and that feeding fish oil to mice for 15 days increases the postprandial plasma adiponectin concentration by two- to threefold, particularly through PPARγ.^44^ In humans, supplementation with 4 g per day of ω-3 fatty acids for 8 weeks increases adiponectin fasting serum levels in patients with coronary artery disease,^47^ and an acute intake of a walnut-enriched meal, which is a good source of ω-3 polyunsaturated fatty acid, also increases postprandial adiponectin release compared with butter- or olive oil-rich meals in healthy young adults.^48^

It is important to emphasize that our results provide evidence that evaluating only fasting adipokine levels does not necessarily reveal adipose tissue dysfunction and that postprandial adipokine levels are required as dynamic indicators of adipose tissue functionality; this is similar to what has been observed for serum glucose concentrations in prediabetic subjects, in whom impaired glucose tolerance has a higher sensitivity than impaired fasting glucose for predicting progression to T2D.^49, 50^

In conclusion, the type of dietary fat consumed for a short period in an adequate proportion had a minimal or no effect on the serum levels of adiponectin or other adipokines during fasting or the postprandium. In contrast, consuming a HFD for a few weeks modified the pattern of adipokines secretion, particularly that of adiponectin. Thus, a HFD containing coconut oil, which is predominantly composed of saturated lauric and myristic acids, decreased adiponectin and resistin secretion and increased leptin secretion. A high consumption of safflower oil, which mainly consists of monounsaturated oleic acid, increased serum RBP-4 and resistin levels but decreased serum adiponectin levels. Interestingly, the HFD with soybean oil, which primarily contains polyunsaturated linoleic and α-linolenic acids,^26^ induced modest changes in the serum adipokine concentration. Further studies are required to understand the molecular mechanisms by which the type of dietary fatty acid regulates the secretion of adipokines during the postprandium.

## Figures and Tables

**Table 1:** Body weight, food intake and biochemical characteristics

	*Coconut oil*		*Safflower oil*		*Soybean oil*	
	*CD*	*HFD*	*CD*	*HFD*	*CD*	*HFD*
Initial weight (g)	191.7±2.91	191.7±2.34	187.5±2.89	194.7±2.33	188.9±3.00	194.9±2.26
Final weight (g)	251.7±3.31	266.7±3.25^**^	243.9±2.91	258.3±2.39^***^	253.2±3.03	268.6±3.63^**^
Food intake (kcal per day)	54.15±3.31	60.04±3.70	54.75±3.43	61.34±3.60	55.87±3.43	62.68±3.78
Fat intake (kcal per day)	8.637±0.52	24.40±1.50^***^	8.732±0.54	24.93±1.46^***^	8.930±0.55	25.47±1.53^***^
Fasting glucose (mg dl^−1^)	139.9±3.56	135.4±3.54	134.9±2.90	134.8±2.56	128.1±3.01	138.3±4.37
Fasting insulin (ng ml^−1^)	0.4235±0.04	0.2504±0.01^**^	0.2671±0.02	0.3113±0.03	0.3175±0.05	0.3811±0.07
HOMA-IR	3.49±0.39	2.11±0.19^**^	2.23±0.21	2.59±0.29	2.44±0.41	3.06±0.59
Fasting triglycerides (mg dl^−1^)	71.27±5.13	54.98±2.82*	58.39±3.22	36.29±4.85^**^	53.36±3.04	34.00±3.32^***^
Fasting cholesterol (mg dl^−1^)	62.56±2.40	63.78±3.09	70.08±3.70	68.29±3.95	59.20±1.96	67.82±4.63

Abbreviations: CD, control diet; HFD, high-fat diet; HOMA-IR, homeostatic model assessment of insulin resistance.

The data are presented as mean±s.e.m.

Significant differences at *P<0.05, **P<0.01 and ***P<0.001 between rats fed the CD and those fed the corresponding HFD containing each dietary oil.
