# Supplementary material for: Fasting and postprandial regulation of the intracellular localization of adiponectin and of adipokines secretion by dietary fat in rats
Source: Nutr Diabetes. 2015 Nov 30;5(11):e184–. doi: 10.1038/nutd.2015.34 (PMC4672355; doi:10.1038/nutd.2015.34)
Supplement: Supplementary Table 1 [file nutd201534x1.pdf]

Supplementary Table 1. Composition of experimental diets

	CD (7% dietary fat, 15.91 kcal from fat)			HFD (21% dietary fat, 36.21 kcal from fat)		
**Ingredients (%)**	**Coconut oil[b]**	**Safflower oil[c]**	**Soybean oil[d]**	**Coconut oil[b]**	**Safflower oil[c]**	**Soybean oil[d]**
Casein[a]	20	20	20	20	20	20
Oil	7	7	7	21	21	21
Cornstarch	39.75	39.75	39.75	30.1	30.1	30.1
Maltodextrin	13.2	13.2	13.2	10.26	10.26	10.26
Sucrose	10	10	10	7.78	7.78	7.78
Cellulose	5	5	5	5	5	5
Mineral mixture[e]	3.5	3.5	3.5	3.5	3.5	3.5
Vitamin mixture[f]	1	1	1	1	1	1
Choline citrate[g]	0.25	0.25	0.25	0.25	0.25	0.25
DL-Methionine[g]	0.3	0.3	0.3	0.3	0.3	0.3

[a] "Vitamin free" casein, Harlan Teklad research diets, Madison, WI, USA. Casein amino acid concentration (g/100 g protein): Ala, 2.8; Arg, 3.4; Asp, 6.3; Cys, 0.3; Glu, 20.5; Gly, 1.6; His, 2.5; Ile, 4.7; Leu, 8.2; Lys, 7.2; Met, 1.9; Phe, 4.4; Pro, 9.5; Ser, 5.0; Thr, 3.8; Trp, 1.6; Tyr, 4.7; and Val, 6.0.

[b] Droguería Cosmopolita, México; FA content (%):SFA, 91.03; MUFA, 7.35; PUFA, 1.61 [27].

[c] Oleico, Coral Internacional, México: FA content (%):SFA, 9.32; MUFA, 7; PUFA, 20.37 [27].

[d] Nutrioli, Ragasa Industrias, Mexico; FA content (%):SFA, 16.31; MUFA, 20.19; PUFA, 63.58 [2, 3, 5-8, 10-18, 20-28, 44-62]

[e] Rogers-Harper, Harlan Teklad research diet, Madison, WI, USA.

[f] AIN-93-VX, Harlan Teklad research diet, Madison, WI, USA.

[g] Harlan Teklad research diets, Madison, WI, USA
