# Supplementary material for: Fasting and postprandial regulation of the intracellular localization of adiponectin and of adipokines secretion by dietary fat in rats
Source: Nutr Diabetes. 2015 Nov 30;5(11):e184–. doi: 10.1038/nutd.2015.34 (PMC4672355; doi:10.1038/nutd.2015.34)
Supplement: Supplementary Figure 1 [file nutd201534x2.pdf]

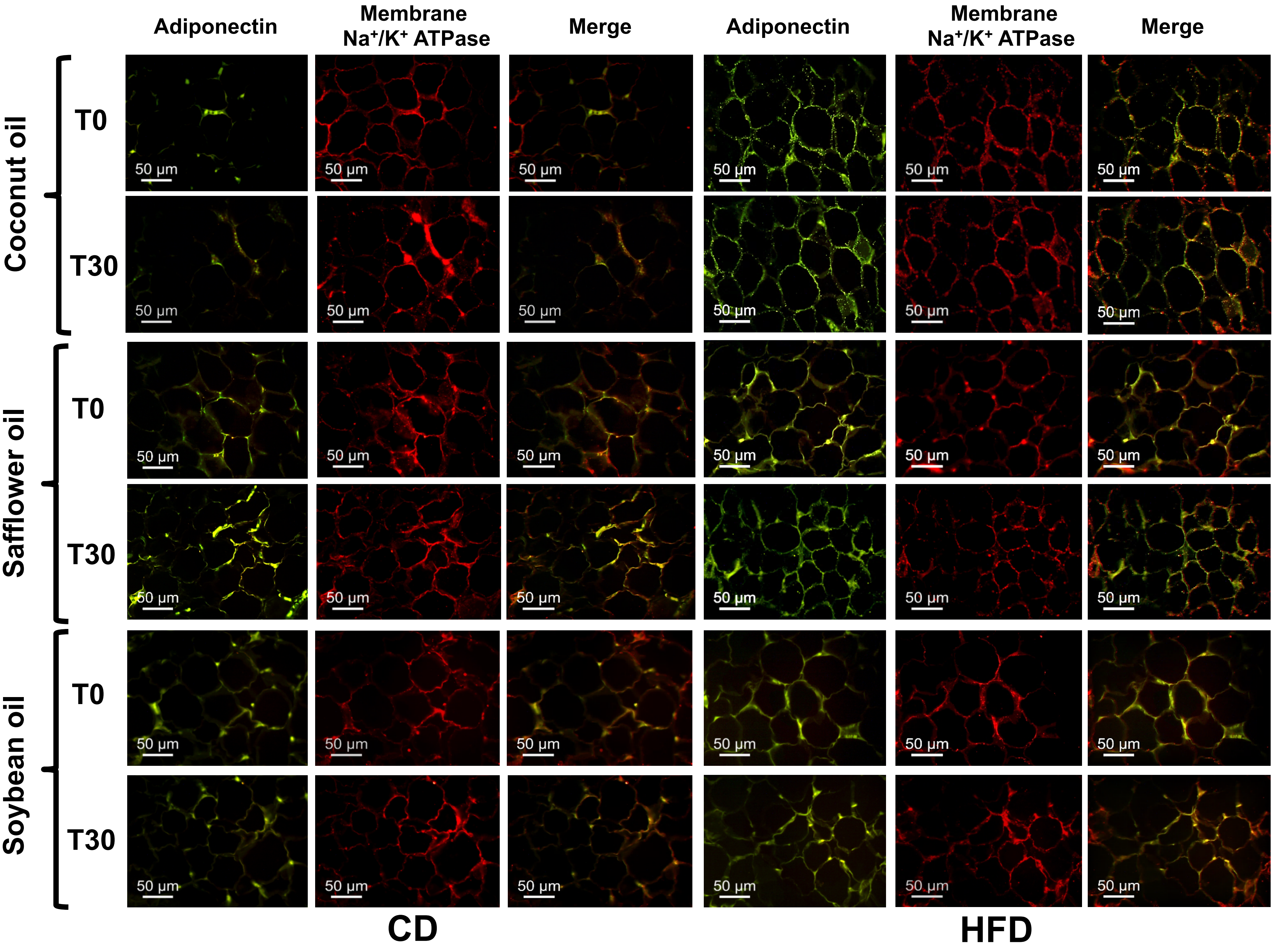

Supplementary Figure 1A) **Representative immunofluorescence images showing the localization of adiponectin (green), Na^+/K^+ ATPase at the plasma membrane (red), and the merged image in epididymal white adipose tissue of rats fed CD or the corresponding HFD containing coconut, safflower, or soybean oil for 21 days, at fasting (T0) and at 30 min after refeeding (T30), captured using a 40X objective. Scale bar 50 microns.**

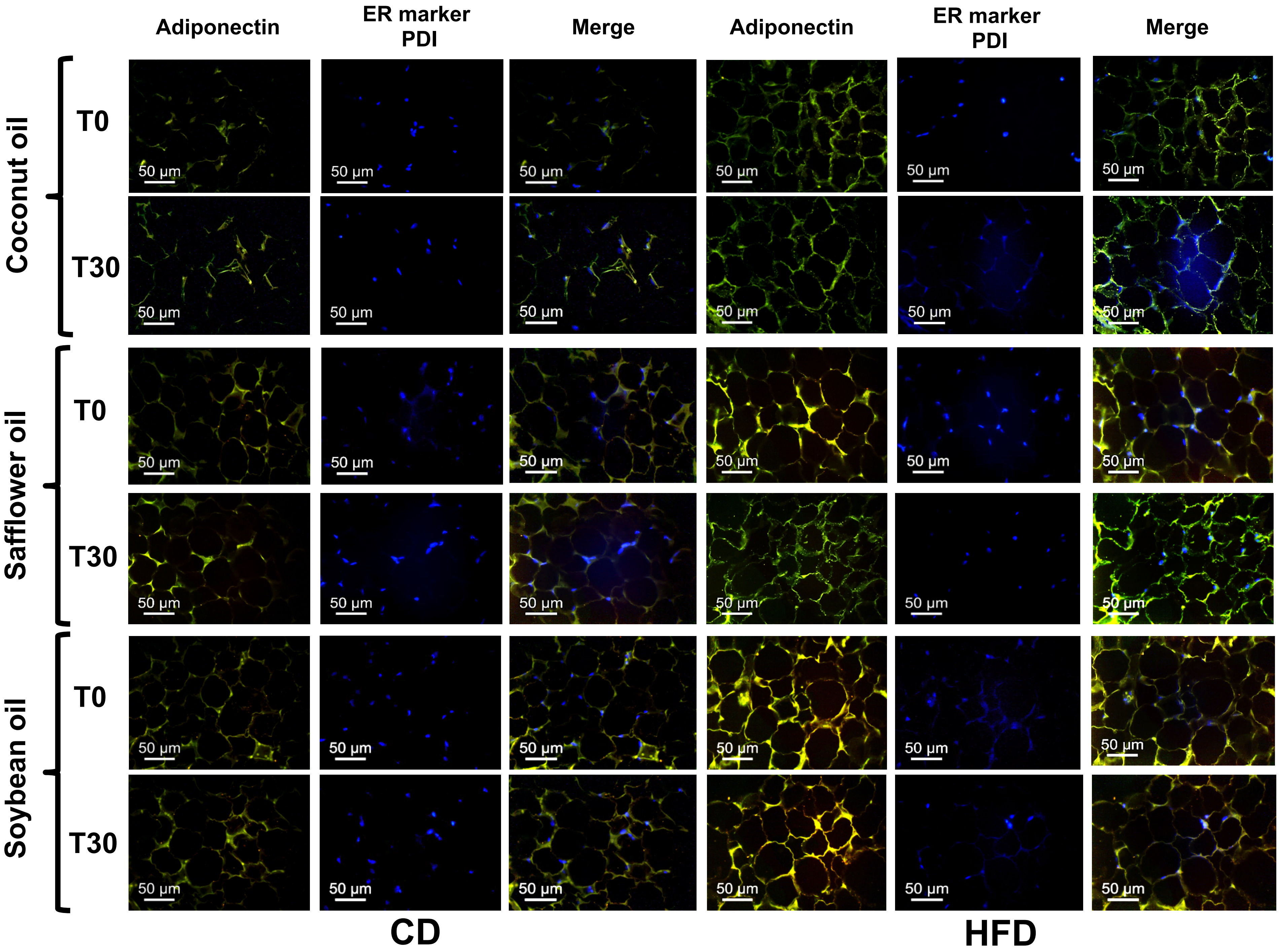

Supplementary Figure 1B) Representative immunofluorescence images showing the localization of adiponectin (green), PDI localized in ER (blue), and the merged image in epididymal white adipose tissue of rats fed CD or the corresponding HFD containing coconut, safflower, or soybean oil for 21 days, at fasting (T0) and at 30 min after refeeding (T30), captured using a 40X objective. Scale bar 50 microns.
