# Supplementary material for: Fasting and postprandial regulation of the intracellular localization of adiponectin and of adipokines secretion by dietary fat in rats
Source: Nutr Diabetes. 2015 Nov 30;5(11):e184–. doi: 10.1038/nutd.2015.34 (PMC4672355; doi:10.1038/nutd.2015.34)
Supplement: Supplementary Figure 2 [file nutd201534x3.pdf]

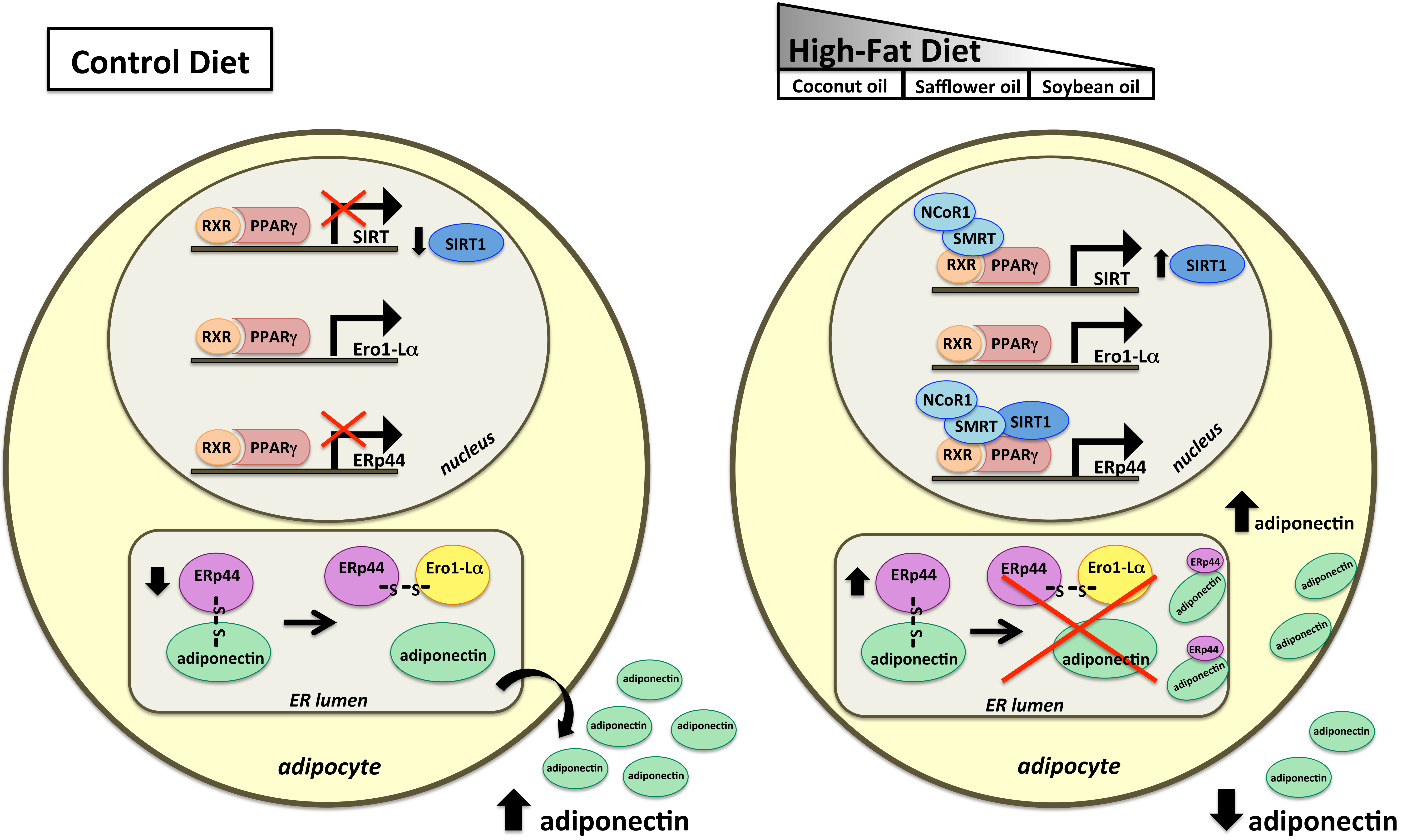

Supplementary Figure 2) Reduction of adiponectin secretion by HFD. In rats fed Control Diet (CD), PPARγ represses the expression of SIRT1 and ERp44 in white adipocytes, and so the release of adiponectin for its secretion from ERp44 occurs through Ero1-Lα, that exchanges the disulfide bond between ERp44 and adipoenctin for a new disulfide bond between it and ERp44 in the lumen of the ER. In animals fed High-Fat Diet (HFD), adiponectin secretion is reduced due to the increment in SIRT1, which in turn represses PPARγ by docking to its negative cofactors (SMRT and NCoR1), inducing in turn the expression of ERp44. High levels of ERp44 induced by HFD, promotes the retention of adiponectin in the inner of adipocytes. The effect is more evident in rats fed coconut oil, then safflower oil and last soybean oil.
